# Prognostic impact of late gadolinium enhancement in patients with high-sensitivity troponin elevation and non-obstructive coronary arteries

**DOI:** 10.1186/1532-429X-17-S1-P175

**Published:** 2015-02-03

**Authors:** Kerryanne Johnson, Boris S Lowe

**Affiliations:** Cardiology, Auckland Hospital, Auckland, New Zealand

## Background

Troponin assays have an established role in the diagnosis and risk stratification of patients with acute coronary syndromes. The lack of an accurate diagnosis and the uncertain prognosis when obstructive coronary disease is absent represents a clinical challenge. We sought to describe the distribution of late gadolinium enhancement (LGE) and its impact on hospital readmission and death in patients with raised high-sensitivity troponin T (hsTnT) not attributed to an acute coronary syndrome.

## Methods

1.5T (n=92) and 3.0T (n=14) cardiac magnetic resonance (CMR) studies and clinical records of 106 patients (mean age 50 years, 37% female) referred by their attending cardiologist were retrospectively assessed. CMR evaluation included left ventricular (LV) volumes and LGE in all patients at an average of 10 days of first hsTnT elevation (≥15ng/L). Patients with obstructive coronary disease (stenosis ≥50%) by coronary angiography were excluded. Hospital readmission and all-cause mortality one-year following the index CMR were assessed.

## Results

The most common presenting symptoms were chest pain (n=67) and dyspnea (n=38); 11 patients presented with cardiac arrest. Presenting ECG documented ST elevation (n=30), ST-segment depression (n=19) and T-wave inversion (n=32). The mean peak hsTnT was 735ng/L (15-15000ng/L). The lowest hsTnT level associated with the presence of LGE was 15ng/L. 83 of 106 (79%) patients underwent coronary angiography. The mean indexed LVEDV 94mls/m2 (42-243) and mean LVEF 54% (15-77). LGE was present in 69 of 106 CMR studies with a midwall/subepicardial distribution most common (n=50). Myocarditis was the most common final clinical diagnosis (n=39), 77% (n= 30) with LGE.

Clinical follow-up data was available for 103 patients. There were 36 hospital admissions in 23 patients for reasons including chest pain (n=9), arrhythmia/palpitations (n=9), heart failure (n=2) and recurrent myocarditis (n=2). 17% (n=12) of patients with LGE had a hospital readmission compared to 27% (n10) of patients who did not have LGE. One patient with LGE and one without LGE died from non-cardiac causes.

## Conclusions

CMR does not yield abnormal LGE in one-third of patients with raised hsTnT not attributed to acute coronary syndromes. One in four patients without LGE were readmitted within one year and this rate is similar to patients with LGE. Newer CMR techniques to evaluate for myocardial edema, fibrosis and necrosis with higher sensitivity are needed.

## Funding

N/A.

**Table 1:** Table 1

Predominant LGE pattern	Number (n=69)
Subendocardial	8
Midwall/subepicardial	50
Transmural	9
Pericardial	2

